# Making products available among community health workers: Evidence for improving community health supply chains from Ethiopia, Malawi, and Rwanda

**DOI:** 10.7189/jogh.04.020405

**Published:** 2014-12

**Authors:** Yasmin Chandani, Sarah Andersson, Alexis Heaton, Megan Noel, Mildred Shieshia, Amanda Mwirotsi, Kirstin Krudwig, Humphreys Nsona, Barbara Felling

**Affiliations:** 1 JSI Research & Training Institute, Inc., Nairobi, Kenya; 2 JSI Research & Training Institute, Inc., Arlington, VA, USA; 3 Ministry of Health Malawi, Lilongwe, Malawi

## Abstract

**Background:**

A UNICEF review of the challenges to scaling up integrated community case management (iCCM) found that drug shortages were a common bottleneck. In many settings, little thought has gone into the design of supply chains to the community level and limited evidence exists for how to address these unique challenges. SC4CCM’s purpose was to conduct intervention research to identify proven, simple, affordable solutions that address the unique supply chain challenges faced by CHWs and to demonstrate that supply chain constraints at the community level can be overcome.

**Methods:**

SC4CCM selected three countries to implement supply chain innovations and developed a theory of change (TOC) framework for the learning phase, which identified the main drivers of product availability and was used for baseline assessments, design, implementation and evaluation of interventions in Ethiopia, Malawi, and Rwanda. Interventions were developed in each country and tested over 12–24 months. Mixed–method follow up assessments were conducted in each country in 2012–2013. The Supply Chain for Community Case Management (SC4CCM) Project then simplified the TOC into a Community Health Supply Chain (CHSC) framework to enable cross country analysis

**Results:**

The findings from interventions in the three countries suggest that the greatest supply chain benefits are realized when all three CHSC framework elements (data flow, product flow, and effective people) are in place and working together. The synergistic effect of these three elements on supply chain performance was most effectively demonstrated by results from the Enhanced Management and Quality Collaborative interventions in Malawi and Rwanda, respectively, which were characterized by lower mean stockout rates and higher in stock rates on day of visit, when compared to other interventions.

**Conclusions:**

Many conditions are necessary to ensure continuous product availability at the community level, however a supply chain works best when three key elements (product flow, data flow, and effective people) are deliberately included as an integral part of the system design. Although these elements may be designed differently in different settings, streamlining and synchronizing them while ensuring inclusion of all components for each element improves supply chain performance and promotes product availability at the community level.

A UNICEF review of the challenges to scaling up integrated community case management (iCCM) conducted in six countries found that drug shortages were one of the most frequently reported bottlenecks and were evident during the implementation and scale–up stages of iCCM [1]. Low or no product availability has even been shown to lead to delays in implementation of iCCM. One of the main conclusions of the March 2014 iCCM Evidence Review Symposium was the need to reduce stock outs in order to increase uptake of iCCM services [2].

Public health supply chains, of which the community is part, generally face chronic challenges in the areas of human resource capacity and skills, general management/management of processes, communication between levels, budget planning, physical infrastructure and capabilities and resources (including storage and distribution capacity), availability and use of data for management decisions, commitment and motivation, and accountability [3]. Each of these elements plays a role in disrupting the availability of essential medicines throughout the supply chain.

These problems are magnified at the community level, as a result of the unique challenges faced by community health workers (CHWs). CHW programs vary widely, but CHWs generally work in remote, rural locations characterized by difficult geographies. Transit to resupply points can be long and difficult and CHWs typically have limited transportation options, given the terrain; often they are forced to use non–motorized forms of transportation such as bikes, donkeys, camels, mules, boats, and even foot [4–6]. Public transport is uncommon and costly. Often, CHWs are not highly literate – m which can cause challenges around recording, reporting, and submitting data – and often have no dedicated facility to work from. Medicines are often stored in drug boxes along with paperwork, and storage space is limited, potentially compromising the quality and security of product storage. CHWs in many countries are unpaid [7], increasing the need for motivation of these workers, especially with regard to supply chain (SC) tasks, which are often seen as tedious, time consuming, and burdensome. Finally, given that CHWs are at the end, or “last mile” of the supply chain, they have no platform for advocacy, so when shortages of essential medicines occur in the system, CHWs tend to miss out on supplies.

In many settings, little thought has gone into the design of supply chains to the community level – community health supply chains have not been deliberately designed to address the unique circumstances of CHWs [8]. Furthermore, limited evidence exists for how to address these unique challenges with a view to improving community health supply chain performance and product availability. The Supply Chain for Community Case Management (SC4CCM) Project’s purpose was to conduct intervention research to identify proven, simple, affordable solutions that address the unique supply chain challenges faced by CHWs and to demonstrate that supply chain constraints at the community level can be overcome. SC4CCM’s mandate was limited to strengthening the community level of the supply chain and did not include funding for commodity procurement. The project’s intent was to gather evidence on “game changing” interventions for ensuring product availability among CHWs, with the goal of helping countries achieve Millennium Development Goal (MDG) 4: Reduce Child Mortality.

This paper presents evidence from community health supply chain innovations implemented in the three project countries that confirm product flow, data flow and effective people as elements that need to be deliberately incorporated into design and which need to work together to effectively improve supply chain performance and the availability of life–saving medicines among CHWs.

## Program description and country context

SC4CCM selected three countries, using these criteria: existence of policies enabling CHWs to deliver the full package of iCCM services (including permission to treat pneumonia with antibiotics); existence of a diverse array of CHW profiles (volunteer vs paid; untrained, limited training, or extensive training); a basic minimum level of procurement for community–level products; and a country context in which JSI was familiar with the overall public health supply chain and its functionality. **Table 1** shows basic demographic and community health statistics for the selected countries.

**Table 1:** Selected data on population, health worker coverage, and iCCM for intervention countries

Characteristic	Malawi	Rwanda	Ethiopia
Population (thousands) (2012)*	14 573	10 537	84 838
Population, percentage rural (2010)**†**	80	81	83
Community and traditional health worker density (per 1000 population)*	0.732 (2008)	1.415 (2004)	0.364 (2009)
Community health policy with full iCCM package‡	2006	2008	2010 (pneumonia added)
iCCM implementation commenced§	2008	2008	2011
CHW name and profile (paid/unpaid, training duration etc)#	Health Surveillance Assistant (HSA). Paid cadre. Initial 12 weeks training in preventive health including primary health care, the EHP, community assessment and mobilization, the role of the VHC, CBHC, WASH, common diseases, patient follow up, and health education. Follow on trainings cover family planning, pre and postnatal care, immunization, nutrition, growth monitoring, iCCM, infection prevention and universal precautions.	Community Health Worker (CHW)/binomes. Volunteer cadre with performance paid based on results, and grouped in cooperatives with start up capital since 2008. 4 weeks training in primary health care services specializing in family planning and iCCM as well as providing information and education on the importance of pre and postnatal care, and other programs including CBP, CBNP, Immunization, DOT, NCDs.	Health Extension Worker (HEW). Paid cadre. 10 months training in environmental sanitation; health and nutrition education; pre and postnatal care; family planning; child health including immunization and iCCM; community mobilization.
Number of CHWs nationwide who manage iCCM products¶	3746	30 000	30 000
Number (and types) of products managed per CHW on routine basis (2010)**	Up to 19 (iCCM, FP, HIV)	~ 6–12 (iCCM, and/or FP)	50+ (iCCM, family planning (FP), HIV, vaccines, other essential medicines)

CBHC – community based health clinic, CBNP –community based nutrition program, CHW – community health worker, DOT – directly observed therapy for tuberculosis, EHP – essential health package, FP – family planning, HEW – health extension worker, HIV – human immunodeficiency virus, HSA – health surveillance assistant, iCCM – integrated community case management, NCDs – neglected communicable diseases, VHC – village health clinic, WASH – water sanitation and hygiene

*Source: Republic of Rwanda National Institute of Statistics Rwanda: 2012 Population and Housing Census. Report on the Provisional Results, November 2012.

†WHO Global Health Observatory.

‡‘Full iCCM package’ defined as CHWs providing treatment for uncomplicated pneumonia, diarrhea, and malaria in children under five. Sources: Ethiopia National Implementation Plan for Community–based Case Management of Common Childhood Illness; IMCI Approach Policy For Accelerated Child Survival and Development in Malawi. 2006; Rwanda National Community Health Policy, 2008, respectively.

§Source: USAID Malawi Community Case Management Evaluation, May 2011, key informant interviews.

§Soure: Advancing Partners in Communities Community Health Systems Catalog.

¶Source: UNICEF 2013 iCCM Survey.

**Source: Advancing Partners in Communities Community Health Systems Catalog and key informant interviews.

**Figure 1:**
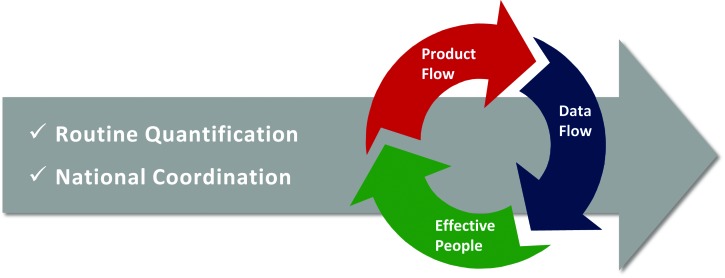
Community Health Supply Chain Framework (A simplified theory of change framework for strengthening the supply chain for iCCM).

**Figure 2:**
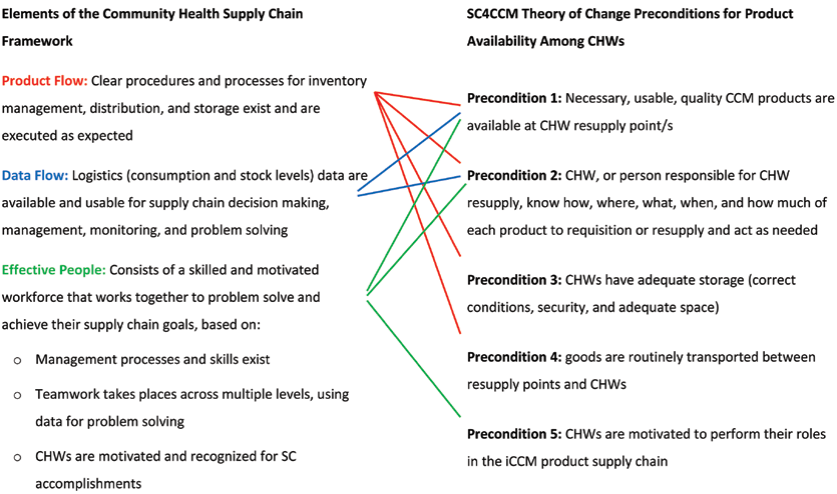
Mapping elements of the Community Health Supply Chain Framework to SC4CCM Theory of Change Preconditions.

**Theory of change framework.** SC4CCM developed a project theory of change (TOC) as a common framework for the learning phase, which identified the main drivers of product availability at the community level and the interrelationships and linkages between these drivers as well as those between the community and higher levels of the supply chain. The TOC identified five preconditions for the main outcome of interest (product availability among CHWs), and served as a framework for design, implementation and evaluation of interventions, providing core indicators for design and implementation of baseline assessment surveys conducted in 2010, in Ethiopia, Malawi, and Rwanda. Baseline findings confirmed the validity of the drivers, interrelationships, and linkages in the TOC, and allowed formulation of three country-specific TOCs [8]. Following the results of the follow up evaluation survey, SC4CCM simplified the TOC into the Community Health Supply Chain (CHSC) Framework, as presented in **Figure 1**, to categorize the necessary preconditions into the basic elements of product flow, data flow and effective people, validated as important for community supply chains. The relationship between the TOC preconditions and the CHSC Framework is presented in **Figure 2**. The CHSC Framework enabled cross–country analysis as well as a demonstration of the interdependency of the three basic elements in enhancing supply chain effectiveness – vital information going into the scale–up phase.

*Product flow* describes how CHWs are resupplied – for example, using a demand–based system or a fixed–quantity supply – and requires clear procedures and processes for inventory management, distribution, and storage.

*Data flow* ensures that logistics (consumption and stock level) data are available and usable for supply chain decision making, management, monitoring, and problem solving. Data flow solutions incorporate mechanisms to capture logistics data at the lowest levels of the system and transmit it in a disaggregated form so that it can be useful for management and decision making. Data flow and product flow are interconnected as the correct data must be collected and visible to the right people to inform product flow.

*Effective people* refers to the workforce involved in making sure product flow and data flow happen; effective people ensure continuous use and improvement of SC skills and practices at the lower levels of the system, build district leadership and ownership for tackling community health SC problems rather than waiting for solutions from higher–level managers, and recognize CHW achievements.

Effective people encompasses:

• *Management processes and skills,* including clear standard operating procedures (SOPs), roles and responsibilities, and provision of SC training/skill and knowledge building,

• *Teamwork,* using a formal structure across multiple levels and/or tools to facilitate group problem solving toward common objectives,

• *Motivation and recognition of CHWs* for SC accomplishments. CHWs take on their responsibilities because they want to serve their community, but often they will need to be motivated to take on SC tasks.

Effective *national–level coordination* and *routine quantification* are fundamental keystones for continuous product availability by ensuring funding for and the timely procurement and distribution of medicines. Well–functioning systems include routine mechanisms for quantification, regular updates to forecasts and supply plans, and close coordination between Ministry of Health (MOH) programs, donors, quantification teams, and procurement units to ensure continuous product availability and to maximize the use of limited resources. In particular, routine quantification and coordination are necessary for ensuring continuous availability of products at the resupply points for CHWs, which in turn is a prerequisite for ensuring that CHWs have products. While we present evidence that product flow, data flow, and effective people will help programs maximize community health supply chain *performance,* quantification and coordination ensure that there are *products available* to flow through the supply chain so that performance can be improved.

Our presentation of results in this paper will focus mainly on the evidence related to the CHSC Framework, since the project did not have a mandate to fully participate in all aspects of quantification and procurement, thus limiting our available evidence.

**Intervention design using theory of change framework and data.** The baseline surveys showed poor combined availability of products required to provide iCCM services among CHWs on the day of visit (DOV) in all countries [8]. **Table 2** shows country findings at baseline. Country–specific results from the baseline surveys were organized according to the original TOC to analyze bottlenecks in the supply chain affecting product availability at the community level. After assembling preliminary results, the project presented findings to in–country stakeholders and representatives of all levels of the supply chain in a series of participatory data validation workshops, which served to both validate results and obtain inputs for designing intervention packages. In all countries, data supported the possibility of testing more than one approach to improving outcomes, but also indicated that intervention packages required a two–phase approach to lay a strong foundation in supply chain knowledge, skills, and procedures before implementing “value–added” innovations. Intervention packages are shown in **Table 3**.The testing period ranged from 12 to 24 months and was characterized by regular monitoring to guide intervention support and adjust the intervention design toward achieving the respective objectives.

**Table 2:** Baseline LIAT survey results, all countries

	Countries		
**Supply chain performance indicator**	**Malawi (No. CHW = 139)**	**Rwanda (No.CHW = 321)**	**Ethiopia (No. CHW = 240)**
**Product availability at community level (DOV)**	• 27% of CHWs had 4 key iCCM products on day of visit (cotrimoxazole, ORS, ACTs 1 × 6 and ACTs 2 × 6) • 35% of CHWs had 3 key iCCM products on day of visit (cotrimoxazole, ORS, and either ACTs 1 × 6 or ACTs 2 × 6)	• 49% of CHWs had 5 key iCCM products on day of visit (amoxicillin, ORS, zinc, Primo Rouge [ACT 1 × 6] and Primo Jaune [ACTs 2 × 6])	• 24% of CHWs had 5 tracer iCCM and FP products in stock on day of visit (ORS, RUTF*, COCs*, DMPA*, and any ACT) (Zinc and cotrimoxazole introduced after baseline)
**Product flow**	Demand–based resupply but using non–standardized forms and data not consistently used for resupply: • 56% of HC staff determined resupply quantities using a standard formula, though 10% used the same quantity as last month, 5% used knowledge from past experience, 5% used another method, and 23% did not know. Transportation is a constraint for CHWs in collecting products: • 18% of CHWs identified a transport related challenge as their number one challenge with collecting and receiving supplies The problems included “it was too long to reach the resupply point,” “there was no transport available,” “the transport was always broken” and “difficulties carrying supplies.”	Unstructured approach with no defined rules or process to drive resupply: • 62% of HCs resupplied based on (non–standard) documentation; 19% of HCs used a variety of (“other”) methods; 8% of HCs provided the same as last month; 7% and 4% of HCs “didn’t know” or used a formula, respectively	Transitioning to a demand–based system, Integrated Pharmaceutical Logistics System (IPLS), but using fixed–quantity supply (kits): • More than 50% of CHWs reported submitting requests when stock runs low or when they stock out • 66% of CHWs report getting their health products from the HC, 44% report getting from the district health office
**Data flow**	Despite the existence for SC procedures, visibility of CHW logistics data was poor at higher levels: • 43% of CHWs reported to HCs using a standard form • 55% of HC staff across ten districts (n = 73) reported HSA supply chain data up to district level, and 14% reported this data disaggregated from HC data	Misaligned reporting system, where data flow did not support decision making: • 97% of CHWs received products from HCs, but only 54% of CHWs submitted logistics data to HCs	Due to lack of training and kit system, CHWs were not using the manual IPLS reporting system for iCCM products: • CHWs mentioned 6–7 different reports that they submitted regularly with no single report having more than 30% of HEWs using them. • 14% of CHWs reported using some kind of stock keeping record
**Effective people**	SC procedures existed, including LMIS forms for CHWs, and CHWs were trained but challenges were identified in supervision and motivation: • 50% reported supervision on SC tasks • When asked about job satisfaction, about 20% of HSAs who manage products ranked a ‘2’ or ‘3’ out of ‘5’	No harmonized procedures for determining resupply quantities for CHWs existed: • CHW motivation to travel and collect products threatened by challenges they mentioned with remuneration (40%), transport (27%) and storage (11%)	Low SC knowledge and skills among CHWs and their HCs: • Only 11% of CHWs and 8% of HC staff had received SC training

ACT – artemisin–based combination therapy, CHW – community health worker, COC – combined oral contraceptive, DMPA – Depo Provera, DOV – day of visit, FP – family planning, HC – health center, HC – health center, HSA – health surveillance assistant, IPLS – Integrated Pharmaceutical Logistics System, LMIS – logistics management information system, ORS = oral rehydration solution, RUTF – ready–to–use therapeutic food, SC – supply chain

**Table 3:** Design of country intervention packages

	Definition	Malawi		Rwanda		Ethiopia
**Intervention package**		Enhanced Management (EM), in 3 of 28 districts nationwide*	Efficient Product Transport (EPT), in 3 of 28 districts nationwide*	Quality Collaboratives (QCs), in 3 of 31 districts nationwide†	Incentives for Community Supply Chain Improvement (IcSCI), in 3 of 31 districts nationwide†	Ready Lessons and Problem Solving, in 28 of ~ 765 woredas nationwide‡
**Product flow**	Clear procedures and processes for inventory management, distribution, and storage exist and are executed as expected	cStock: mHealth reporting and resupply system for CHWs	cStock Continuous review inventory control system / bicycle maintenance	Standard Resupply Procedures (RSP)	RSP	Ready Lessons
**Data flow**	Logistics (consumption and stock levels) data are available and usable for supply chain decision making, management, monitoring, and problem solving	cStock	cStock	RSP	RSP	Ready Lessons
**Effective people**	Consists of a skilled and motivated workforce that works together to problem solve and achieve their supply chain goals, based on: • Management processes and skills • Teamwork across multiple levels, using data for problem solving • CHWs motivated and recognized for SC accomplishments	DPATs	None	Teamwork: Quality Improvement Teams (QITs) Motivation: Allowances	Motivation: Allowances and performance–based incentive paid to CHW cooperative	Ready Lessons Problem Solving

ACT – artemisin–based combination therapy, CHW – community health worker, EM – enhanced management, DMPA – depo provera/depot medroxyprogesterone acetate, DPAT – district product availability teams, EPT – efficient product transport, FP – family planning, IcSCI – incentives for community supply chain improvement, mHealth – mobile health, ORS – oral rehydration solution, QIT – Quality Improvement Team, SC – supply chain, QC – Quality Collaboratives, RSP – resupply standard procedures, RUTF – ready–to–use therapeutic food

*Source: CIA World Factbook. Available at: https://www.cia.gov/library/publications/the-world-factbook/geos/mi.html; accessed: 10 November, 2014.

†Source: National Institute of Statistics, Rwanda; 2006. Available at: http://www.statistics.gov.rw/geodata. Accessed: 10 November, 2014.

‡Source: Population and Housing Census Report – Country – 2007. Central Statistical Agency, 2010–2007. Available at: http://www.csa.gov.et/newcsaweb/images/documents/surveys/Population%20and%20Housing%20census/ETH-pop-2007/survey0/data/Doc/Reports/National_Statistical.pdf. Accessed:10 November, 2014.

***1. Malawi.*** At baseline, Malawi was in the process of implementing a demand–based resupply system; however, data was not visible at all levels of the system with only 55% of health center staff across ten districts reporting CHW supply chain data up to district level, and only 14% reported this data disaggregated from health center data; therefore CHW–specific supply chain data was not available for management decision making or performance monitoring at higher levels of the system. Reporting rates were low and few data were available to district managers for identifying and resolving stock outs or other management issues. Given that 89% of the CHWs surveyed at the 2010 baseline had mobile phones, and network coverage was high, the project developed a simple SMS and web–accessible reporting and resupply system, cStock. cStock was intended to improve the resupply process by enhancing communication between CHWs and their resupply points, to facilitate visibility of real–time CHW logistics data at district and central levels, and to enable supply chain managers to respond immediately to performance or product availability issues. The design of cStock mirrors processes for the demand–based resupply system while improving data visibility through better data flow for operations and management, and improving product flow using a streamlined resupply process. cStock was combined with two different approaches (Enhanced Management [EM] and Efficient Product Transport [EPT]), that were tested side by side in three districts each. EM addresses all three framework elements by combining cStock, which addresses product flow and data flow, with the establishment of District Product Availability Teams (DPATs), which aimed to improve the effectiveness of the people by promoting team performance practices through the use of data to inform decisions and improve supply chain performance. DPATs comprise district management, health center staff, and CHWs who have a shared vision and collective commitment to ensuring continuous availability of products through use of data for continuous improvement and recognition of good CHW performance.

The EPT intervention only aimed to address two of the three framework elements, namely product flow and data flow, and did not address the element of effective people. Transport was a big challenge identified at baseline; although the MOH provides all CHWs with bicycles, breakdowns were frequent, reducing CHWs’ ability to collect supplies regularly. In addition to cStock, EPT introduced two approaches to improve product flow. First, a continuous–review inventory control system that allowed CHWs to make more frequent trips to collect smaller amounts of supplies during their scheduled visits to health facilities and reducing the need for them to make special trips for product pickup. Second, EPT trained CHWs in regular, preventive bicycle maintenance to reduce breakdowns and repairs needed to keep the bicycles functioning.

Malawi conducted an annual quantification that included the iCCM program. Quantification for iCCM is complicated, however, by the fact that all of the products used by CHWs in Malawi are also used at higher levels of the health system or by other programs, requiring the input of good quality data from all programs and levels to develop a robust iCCM program forecast and supply plan. Further, donor support to CHWs in Malawi often targeted individual districts. Thus distribution data and data on products used by level and program were not always available. These circumstances made coordination and monitoring overall stock levels, other than the community level through cStock, difficult.

***2. Rwanda.*** Baseline results demonstrated that CHWs were not resupplied according to any rules. The foundational intervention, therefore, was to establish a demand–based resupply system, called Standard Resupply Procedures (RSPs) for CHWs. The processes targeted the Cell Coordinator (CC) as the primary actor to collect and aggregate data from CHWs in their cell and resupply them with products, to increase efficiency at the health center level. If scaled nationally, RSPs would result in monthly reports for approximately 2150 CCs rather than 30 000 individual CHW reports. The intervention required the use of three basic tools: CHW stock cards to capture consumption data and stock data, a simple tool to calculate resupply quantities (“the magic calculator”), and a resupply worksheet (RSW) that CCs use to aggregate data for all CHWs in their cell each month.

The RSPs were implemented in six test districts and ensured sufficient and appropriate data flow for operations and SOPs as part of the first step toward developing effective people. The hypothesis was that designing supply chain processes and imparting skills were necessary first steps but not sufficient alone to significantly improve product availability. Thus, once RSPs were implemented, and the foundation for product flow and data flow was established, two different strategies (Quality Collaboratives [QCs] and Incentives for Community Supply Chain Improvement [IcSCI]) were tested side by side; both aimed at making CHW, health center and district staff engaged in supply chain tasks more effective and improving product availability.

The QC approach, previously used successfully to solve bottlenecks in clinical work (9], involves establishing and training Quality Improvement Teams (QITs) at health centers to find solutions for operationalizing the new resupply procedures at the CHW level. The aim of the QCs, or networks of QITs, is to close the gap between desired and actual performance by using data to target and address problems, and then developing, testing/implementing, and spreading changes quickly across many teams and/or organizations. QITs brought CCs, health center, and district staff together as a team to look at data on SC performance collected by CCs on supervision checklists; problems were identified and prioritized; action plans were developed and progress tracked. The project worked closely with the MOH to ensure the QITs functioned as expected during the testing period.

In contrast, IcSCI aims to strengthen the commodity supply chain by adding supply chain related–indicators to Rwanda’s existing community performance–based financing scheme for CHWs, which targets improvements in delivery of health services at the village level. IcSCI provides an incentive package that specifically rewards CHWs for improved performance of supply chain tasks linked to nine supply chain indicators by providing monetary incentives to CHWs through their community cooperatives based on quarterly performance scores. In essence, both QCs and IcSCI targeted all three elements of the framework, namely product flow, data flow and effective people, although QCs addressed effective people more comprehensively than IcSCI by including a formal teamwork component, which was more informal and indirect in the incentives approach.

In Rwanda, the community health desk within the MOH plays a strong role in coordination, ensuring that quantification in collaboration with different programs (Malaria, MCH, etc) takes place annually and supply plans are monitored regularly, as well as providing funding for product procurement for iCCM and working with the national procurement unit (Medical Procurement and Production Division) to coordinate procurement of products, many of which are used exclusively at the community level.

***3. Ethiopia.*** Baseline results showed that CHWs and health centers were ineffective in managing health products because they lacked SC knowledge and skills. The Federal Ministry of Health (FMOH) and donors were supplying iCCM products to CHWs using a fixed–quantity supply (FQS) method: kits. The national logistics system in Ethiopia is transitioning to a new demand–based supply chain system (the Integrated Pharmaceutical Logistics System, or IPLS); hence, SOPs existed for all levels, although they had not been fully implemented at any level of the system. The IPLS outlines how data and products should flow between the levels of the supply chain, so the project’s priority was to test a way of rapidly and affordably building foundational CHW supply chain knowledge and skills around the SOPs for IPLS, as a first step toward addressing the effective people element, with the expectation that the approach could be scaled up to all 30 000 plus CHWs, who could then move away from fixed quantity supply to a demand–based system. Three different approaches were taken to implement the training using existing activities at health centers as opportunities to impart SC knowledge and skills. Two approaches used a group training method during monthly meetings at the health center, and one approach used one–on–one training or on the–job–training (implemented by another project and called the comparison group) at the time HEWs came to collect products. In the two groups that used the group training method, one group received follow up support (intensive group) and the other received no additional support (non–intensive group). Key supply chain skills for CHWs were distilled into five one–hour “Ready Lessons” that could be administered in any order and/or repeatedly; these lessons were combined with supply chain problem solving to address bottlenecks and identify gaps in skills that needed to be addressed. While recognition/motivation is a key element of “effective people,” this element was deliberately excluded from the design since “Ready Lessons” were intended to be administered during pre–existing meetings that already had HEW recognition on the agenda. Because of Ethiopia’s vast geography and large numbers of CHWs, the 2010–2013 period was spent implementing and testing the foundational intervention, with the “added–value” intervention planned for 2013–2015 (not included in this paper). Supply chain knowledge and skills are critical prerequisites for operationalizing IPLS; however, training is necessary but not sufficient for developing “effective people” or for significantly improving product availability. Therefore, significant improvements in product availability and other key supply chain indicators were not expected from this intervention.

Ethiopia conducted regular quantification for iCCM but faced additional challenges with coordination because of the kit system, as six months of supply for each site had to be available in fixed quantities for kitting centrally before distribution. Given the different funding and procurement cycles of the government and various donors, the required level of coordination was difficult to achieve. Additionally, as the quantities in the kits were based on initial estimates of need that were not revised in light of actual consumption patterns, CHWs ran out of some items rapidly while others lasted much longer than anticipated.

## Methods

Using the TOC as the guiding evaluation framework, the project conducted baseline and follow up assessments in select areas of the three countries in 2010 and 2012–2013, respectively, using complementary quantitative and qualitative methods. The quantitative survey tool, called the Logistics Indicator Assessment Tool (LIAT), was adapted from tools originally developed by the USAID | DELIVER PROJECT, including questionnaires, inventory assessment forms, storage assessment forms, and key informant interview guides [9,10]. The survey was tailored to each level of the supply chain, from central medical stores down to the community level, to capture processes, behaviors, and product availability along each step in the chain, and to measure indicators of intervention implementation. Tools were field–tested and adapted for each country setting [11]. Permissions for the assessments were obtained from all relevant MOH partners and institutional review board (IRB) approval was obtained in Malawi and Rwanda, where it was required.

Survey samples were not intended to be nationally representative, but rather chosen to first diagnose major iCCM supply chain strengths and weaknesses in a cross–section of districts served by key iCCM partners, and then to follow the supply chain from the central level to the community level. Purposeful selection at the district level was done based on existence of a functioning iCCM program, geographic variation, and balance of iCCM partner support. Probability proportional to size sampling was used to randomly select health facilities and CHWs at the lower levels of the supply chain. In all countries, CHWs were the unit of analysis. **Table 4** shows the full details on survey dates, sample sizes, and levels of the supply chain visited by country surveys.

**Table 4:** Evaluation profile: dates, sampling, and intervention grouping, by country

	Malawi			Rwanda				Ethiopia		
**Evaluation dates:**										
Baseline (BL)	May – June 2010			Sept – Nov 2010				July – Sept 2010		
Follow up* (FU)	Jan – Mar 2013			Apr – May 2013				Oct – Dec 2012		
Intervention kickoff and duration of testing period	EM and EPT training (June – Dec 2011) Monitoring and Intervention support (Jan 2012 – Feb 2013)			RSPs (Aug 2011 – March 2013) QCs (April 2012 – March 2013) IcSCI (April 2012 – March 2013)				Ready Lessons/Problem Solving TOTs for HCs (Oct – Dec 2011) Assumed rollout to HEWs (Jan – June 2012)		
**Overall LIAT sample:**										
Districts FU (BL)	10 (10) of 28 nationwide†			10 (10) of 31 nationwide‡				28 (26) Woredas of ~ 765 woredas nationwide§ 12 (9) Zones of ~ 85 nationwide§		
Health Centers FU (BL)	76 (77)			108 (100)				82 (74)		
CHWs FU (BL)	249 (249)			349 (321)				263 (245) Health Posts		
**LIAT sample size by intervention group:**										
	**EM**	**EPT**	**Comparison**	**QCs**		**IcSCI**	**Comparison**	**Intensive**	**Non–intensive**	**Comparison OJT**
District/Woreda FU (BL)	3 (3)	3 (3)	4 (4)	3 (3)		3 (3)	4 (4)	8 (8)	10 (9)	10 (9)
Health Centers FU (BL)	25 (26)	23 (25)	28 (26)	31 (30)		37 (31)	40 (39)	24 (28)	30 (20)	28 (26)
CHWs FU (BL)	81 (81)	78 (83)	90 (85)	105 (85) 70 (0) CCs		116 (102) 78 (0) CCs	128 (134)	80 HPs (69)	92 (102)	91 (74)
**% CHWs managing iCCM products:**										
BL	139 of 249 (56%) manage any health products (including iCCM, FP, HIV)				65%of 321 manage amoxicillin 250mg, ORS, zinc 20mg, Primo Rouge (ACT 1 × 6), Primo Jaune (ACT 2 × 6)			71 of 245 (29%) manage ORS, RUTF any ACT, COCs, and DMPA		
FU	100% of 249 manage cotrimoxazole 480mg, both LA (1 × 6 and 2 × 6), and ORS				94% of 349 manage amoxicillin, 150mg, ORS, zinc 10mg, Primo Rouge (ACT 1 × 6), Primo Jaune (ACT 2 × 6)			151 of 263 (58%) manage ORS, RUTF, any ACT, COCs, and DMPA		

ACT – artemisin–based combination therapy, BL– baseline, COC – combined oral contraceptives, CHW – community health worker, DMPA – depo provera/depot medroxyprogesterone acetate EM – enhanced management, EPT – efficient product transport, FP – family planning, FU – follow up, HEW – Health Extension Worker, HC – health center, HIV – human immunodeficiency virus, iCCM – integrated community case management, IcSCI – Incentives for Community Supply Chain Improvement, LA – artemether/lumefantrine, LIAT – logistics indicator assessment tool, OJT – on the job training, ORS – oral rehydration solution, QC – Quality Collaboratives, TOT – training of trainer, RUTF – ready-to-use therapeutic food

*Follow up survey results also referred to as Follow Up survey in Tables 5, 6 and 8 and text.

†Source: CIA World Factbook (https://www.cia.gov/library/publications/the-world-factbook/geos/mi.html). Accessed: 10 November, 2014.

‡Source: National Institute of Statistics, Rwanda; 2006. Available: http://www.statistics.gov.rw/geodata. Accessed: 10 November 2014.

§Source: Population and Housing Census Report – Country – 2007. Central Statistical Agency, 2010–2007. Available at: (http://www.csa.gov.et/newcsaweb/images/documents/surveys/Population%20and%20Housing%20census/ETH-pop-2007/survey0/data/Doc/Reports/National_Statistical.pdf. Accessed: 10 November, 2014.

Quantitative data for both surveys and all three countries were collected by local evaluation partners, all selected through competitive processes. Enumerators were trained to interview CHWs and other staff managing supplies of medicines, conduct product inventories, and rate storage conditions. Data collectors used Nokia e71 and e63 smartphones loaded with DataDyne’s Magpi application, which allowed for streamlined data entry and immediate review of data after uploading records to a web–based system. Qualitative methods were also employed for deeper understanding of user experiences, but this paper focuses primarily on quantitative results.

In Malawi and Rwanda, supplemental data sources were also considered, including routine data collected through cStock and the IcSCI indicators database respectively. In Malawi, routine logistics monitoring data submitted by CHWs using cStock were utilized to study inventory trends over time between the EM and EPT groups. The web–based cStock dashboard provided reports showing monthly stock reporting rates, average time taken to restock the drugs (lead time), product availability, and stock outs for these time periods for the six intervention districts (three for the EM group and three for the EPT group). In Rwanda, the project, over the intervention period maintained quarterly performance scores for the nine supply chain indicators, submitted by health centers, for the IcSCI group (three districts) in the IcSCI indicators database.

### Study Groups and DiD

After the formative assessments in each country in 2010, the project formed three groups from original baseline evaluation areas by matching geographical and demographic characteristics, and other external dimensions including iCCM partner coverage, prevalence of diarrhea, malaria, and cough, as well as baseline CHW iCCM product availability, to create comparable groups. Two of the three groups were randomly assigned a unique intervention, while the third group was assigned as the comparison group. For Malawi and Rwanda, this division of areas was designed to facilitate a difference in difference (DiD) analysis to calculate the effect of the interventions on a key supply chain indicator (CHW product availability) by comparing the average change over time in this indicator for the intervention group to the average change over time for the comparison group. **Table 4** provides further details on and division of areas into intervention and comparison groups. In Malawi and Rwanda, a DiD regression analysis was conducted using iCCM product availability as the outcome variable. Because of the extraordinary challenges related to parallel supply chains in Malawi, the DiD results were inconclusive and further analyses were conducted to determine the effect of the interventions on supply chain performance and product availability. In Rwanda, the DiD analysis attempted to control for factors that may have affected the product availability over time indicator, including formal training of CHWs on the management of medicines and health products, training of CHWs in pneumonia, malaria, or diarrhea, and CHWs reporting transport obstacles in getting to their resupply point (**Table 7**). Limitations to this model include the real possibility that outside factors, applied unequally between groups over the three year period between baseline and follow up surveys, also caused changes, reducing the ability to attribute changes to the interventions alone.

**Table 7:** Rwanda difference–in–differences (DiD) regional results: IcSCI and QC groups

	IcSCI*						QC*					
**Group**	IcSCI		Non–intervention		DiD**†**	N	QC		Non–intervention		DiD**†**	N
**Time**	BL	FU	BL	FU			BL	FU	BL	FU		
Percent of CHWs who manage all **5 products**, in stock on DOV	53	46	58	37	**14**	351	35	62	58	36	**49**‡	346

CHW – community health worker, DiD – Difference in Differences, DOV – day of visit, IcSCI – Incentives for Community Supply Chain Improvement, QC – Quality Collaboratives,

*Controls: CHW has formal training on how to manage medicines and health products, CHW has training in pneumonia, malaria, or diarrhea, CHW has obstacles to transport

†DiD is calculated as DID = (Intervention Follow up% – Intervention Baseline%) – (Comparison Follow up% – Comparison Baseline%). Results displayed represent two steps in the analysis of the data: the significance, denoted by the stars, represents the results from the multivariate logistic regression on the time–group interaction variable, which is the key independent variable of a DiD regression. Since the interaction coefficient is non–intuitive, we have instead depicted the difference over time between the intervention and non–intervention groups using the predicted probabilities resulting from the regression. Essentially, this is the net percentage point change in the intervention region once the comparison group change is subtracted.

‡*P* < 0.001.

### Country analyses

For both baseline and follow up surveys, frequencies and cross–tabulations were carried out using SPSS 18 and STATA version 11. Analyses were conducted using pathways identified in each country–specific TOC [12–14]. Indicators associated with the precondition pathways were laid out to determine progress along the pathway of change, both to validate the TOC as well as identify where obstacles may have prevented achievement of outcomes.

For Malawi, cStock data were retrieved for the 18-month period from January 2012 to June 2013 and average values of key supply chain indicators were calculated. Paired Student’s t–tests were conducted to compare the trends between the EM and EPT groups. For data from the Rwanda IcSCI indicators database, Pearson chi squared tests were run to determine whether there was a significant difference in the performance of CHWs on select indicators by district over the four quarters of the testing period.

### Cross–country analysis

Following completion of follow up surveys in all countries, the project partnered with Accenture Development Partners (ADP) to develop a practical framework to facilitate cross–country analysis and synthesis of intervention findings using a broader lens. SC4CCM and ADP refined the project and country TOCs into the CHSC framework to capture important and consistent results from each country evaluation. Results related to each intervention package were categorized by product flow, data flow, and effective people and interpreted with a view to determining the effectiveness of each package.

Effectiveness was defined as achieving the intended or desired outcome of the intervention. The ultimate goal of any supply chain is improved product availability – to ensure that the service delivery point, in this case the CHW, has usable and quality medicines available to serve clients when needed. However, product availability is influenced by numerous factors, as shown by the TOC. Although supply chain performance is a critical factor, the greatest prerequisite is having products flowing through the national supply chain; supply chain performance is irrelevant if there are no products. The expected outcome for interventions in Malawi and Rwanda consisted of improvements in community supply chain performance, which we hypothesized, would lead to improvements in product availability. Improved supply reliability, defined as reductions in stockout rates, was used as an alternate outcome measure in Malawi due to limitations associated with attributing improvements in product availability to project efforts. Indicators for supply chain performance varied in each country, given the data available. In Ethiopia, the expected outcome was an improvement in supply chain competencies, leading to improvements in key supply chain practices.

## RESULTS

Follow up results for Malawi and Rwanda are presented first according to the CHSC Framework elements of product flow, data flow, and effective people, and then the results of intervention packages as a whole are compared to understand how the combination of the different elements affected the supply chain performance indicators and product availability. In Ethiopia, results are presented based on the aspect of the effective people element only. Data source is the LIAT survey unless otherwise noted, but results draw from focus group discussions (FGDs), cStock dashboard reports and the IcSCI indicators database.

### Malawi

All aspects of the EM intervention were fully implemented, while only the cStock component of EPT was implemented – the continuous review inventory management system was not implemented, with users finding it burdensome, and neither was regular practice of preventive bicycle maintenance by trained CHWs. Since EPT was not designed to address the effective people element, and its product flow design was unchanged, the project used the comparison of key supply chain performance indicators between EM and EPT groups to show the added value of the effective people (DPAT) component to product and data flow (cStock) in the EM group. **Table 5** summarizes key follow up results for Malawi.

**Table 5:** Summary of quantitative follow up survey results, Malawi (source: LIAT survey, unless otherwise noted)

	Definition		EM Group		EPT Group		NI Group*	
**Primary objective**		CHWs have usable and quality essential medicines available when needed for appropriate treatment of pneumonia and other common diseases of childhood		64% of CHWs had all 4 products† in stock on day of visit		59% of CHWs had all 4 products† in stock on day of visit		63% of CHWs had all 4 products† in stock on day of visit	
**Product flow**	Clear procedures and processes for inventory management, distribution, and storage exist and are executed as expected		98% of CHWs reported using cStock, 6% use Form 1A, and 1% use another form for ordering health products from their resupply point (multiple responses allowed)		91% of CHWs reported using cStock, 13% use Form 1A, and 5% use another form for ordering health products from their resupply point (multiple responses allowed)		48% of CHWs reported using Form 1A, 34% use an unspecified request form, and 23% use another form for ordering health products from their resupply point (multiple responses allowed)	
			92% of Drug Store in–Charges reported using cStock, 12% “give as much as I have available,” 8% use Form 1A, and 4% use LMIS 01G to determine quantities to resupply CHWs (multiple responses allowed)		91% of Drug Store in–Charges reported using cStock, 17% “give as much as I have available,” 17% use Form 1A, and 9% use LMIS 01G to determine quantities to resupply CHW (multiple responses allowed)		48% of Drug Store I/Cs reported using Form 1A, 17% use LMIS 01G, 10% reported that they “issue standard amount,” 10% “give as much as I have available,” and 24% reported they use another way to determine quantities to resupply CHWs (multiple responses allowed)	
			Average lead time (request to receipt) for HSAs was 12.8 days from Jan 2012–June 2013‡ (multiple responses allowed)		Average lead time (request to receipt) for HSAs was 26.4 days from Jan 2012–June 2013‡ (multiple responses allowed)		N/A	
**Data flow**	Logistics (consumption and stock levels) data are available and usable for supply chain decision making, management, monitoring, and problem solving		94% of CHWs send reports to HCs monthly from Jan 2012–June 2013‡		79% of CHWs send reports to HCs monthly from Jan 2012–June 2013‡		N/A	
			85% of CHWs submitted complete reports from Jan 2012–June 2013‡		65% of CHWs submitted complete reports from Jan 2012–June 2013‡		N/A	
**Effective people**	Management processes and skills; Teamwork across multiple levels, using data for problem solving; CHWs are motivated and recognized for SC accomplishments		84% of CHW Supervisors reported DPAT meetings were held		N/A		N/A	
			96% of CHW Supervisors reported conducting a DPAT meeting		N/A		N/A	
			100% of District & CHW Supervisors reported finding product availability teams useful		N/A		N/A	

CHW – community health worker, DPAT – district product availability teams, EM – enhanced management, EPT – efficient product transport, HSA – health surveillance assistant, I/C – in charge, LMIS – Logistics Management Information System, NI – non intervention, SC – supply chain

*Comparison group data available for Primary objective and some product flow indicators only, other data points in the table relate specifically to the interventions and are not relevant in the comparison group.

†Products include cotrimoxazole 480 mg, both ACTs (1 × 6 and 2 × 6), and ORS.

**‡**Source is cStock data from Jan 2012 to June 2013; this includes data from 392 HSAs in EM districts and 348 HSAs in EPT districts. Significant differences were seen for all three indicators between EM and EPT results (*P* < 0.001).

**Product Flow.** Follow up evaluation results show that inventory management was streamlined and standardized product flow in both EM and EPT groups, largely due to cStock, compared to the non–intervention (NI) group, where less consistency was shown in forms used to request and resupply. Ninety–eight percent of CHWs in the EM group and 91% in the EPT group reported using cStock for requesting health products, compared to NI, where 48% of CHWs reported using Form 1A, 34% use a request form, 9% use LMIS–01G, and 14% use another form (multiple responses allowed). Additionally, 92% of Drug Store in–Charges in the EM group and 91% in the EPT group reported using cStock to determine quantities to resupply CHWs. The NI group reported using various resupply mechanisms, with 48% of Drug Store in–Charges using Form 1A, 17% using LMIS 01G, 10% reporting that they “issue standard amount,” 10% “give as much as I have available,” and 24% reporting “other.”

**Data flow.** Results also showed improvements in the reporting and visibility of community–level stock logistics data. At baseline, the average CHW reporting rate using a standard form across the ten districts was 43%. Data visibility at higher levels also improved, with logistics data from all cStock–reporting CHWs (94% in EM and 79% in EPT) accessible at district–level through the dashboard. At baseline, while 55% of health center staff reported CHW supply chain data up to district level, only 14% reported sending disaggregated CHW logistics reports to a higher level, resulting in very limited visibility of community data for district level decision makers.

**Effective people.** Follow up findings show a high frequency of DPAT meetings in the EM group and evidence that data was used to monitor and improve supply chain performance and recognize CHW achievements. Eighty–four percent of CHW Supervisors reported that district–level DPAT meetings were held and 96% of CHW Supervisors reported conducting a DPAT meeting at the health center level. Health center staff monitored the performance of the community supply chain using cStock data, with the majority using either reports pulled from cStock (56%) or resupply worksheets (40%) where cStock transactions are recorded. FGDs highlight how DPATs meetings were used to improve key supply chain performance indicators and product availability; as one CHW explained, “*We talk about our reporting rate and how best to improve it, the products.”* Another CHW Supervisor shared that they, *“…discuss the over–stocking or under–stocking and we discuss how we can share the drugs.”*

FGDs highlighted the benefits of the DPATs in improving communication and team work, as described by one CHW, “*We also discuss and encourage teamwork among the medical assistant and us to work together, because when we send stock on hand, we depend on them to respond all the time, and that has enhanced our communication and team work*.” CHWs also described how DPATs motivated them; one CHW shared “s*ometimes when we are in our meeting the medical assistant compliments one of the CHWs and when he does so, we are motivated as well to perform better so that we can be complimented*.”

**Comparing intervention packages.** Comparing the results of EM and EPT in **Table 5** for three key supply chain performance indicators (lead times, reporting rates, and completeness of reporting) demonstrates how the EM group outperformed the EPT group suggesting the difference was likely due to the DPAT – effective people component of EM. These indicators were calculated using cStock data for the period of January 2012 to June 2013 and T–tests yielded significant differences (*P* < 0.001) for all three indicators.

**Figure 3:**
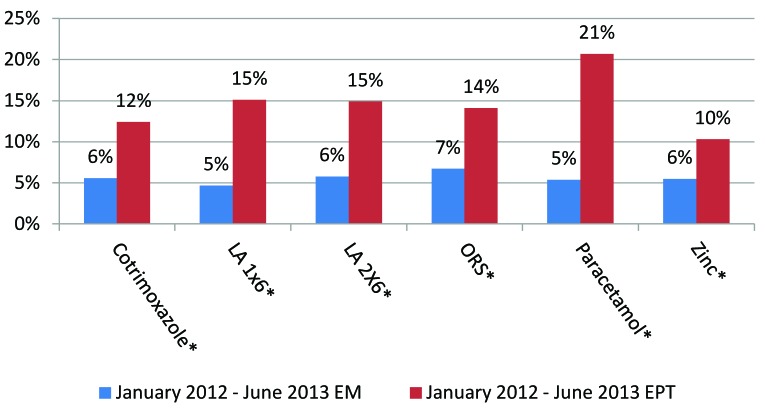
Mean percentage stockout rate over 18 months, by product, for EM vs EPT districts, (January 2012–June 2013). Asterisk indicates *P* < 0.001.

Product availability for four tracer iCCM products on the day of visit more than doubled, increasing from 27% of all CHWs with products at baseline to 64% in EM, 59% in EPT, and 63% in NI at follow up. However, due to the presence of a number of parallel supply chains bypassing the government supply chain to deliver directly to health facilities or CHWs over the testing period, it was not possible to isolate the impact of our interventions from that of the donor–supported drug distribution for this indicator. Therefore, given that product availability data could not be used to evaluate program effectiveness, the project measured supply reliability by comparing stock out rates in cStock over the period of the intervention between EM and EPT groups. EM stock out rates were consistently lower (below 10%) than those for EPT for all six products over the period January 2012–June 2013. Stock out results are presented in **Figure 3** as a measure of mean percent CHW stock out rates by product. These differences were statistically significant at the *P* < 0.001 level for all products. Results suggest higher levels of supply reliability in the EM intervention than the EPT intervention.

### Rwanda

Results show that all aspects of the RSPs, QCs, and IcSCI interventions in Rwanda were well implemented. The design and implementation of RSPs was meant to set the foundation for good product flow and data flow processes for the community level and rationalize the movement of people, data, and medicines. However, because the comparison group had no equivalent role to Cell Coordinator, it is not possible to evaluate improved product and data flow due to RSPs in the intervention groups vs the NI group. **Table 6** provides a summary of key follow up survey results for Rwanda.

**Table 6:** Summary of quantitative follow up survey results, Rwanda (source: LIAT survey, unless otherwise noted)

	Definition	QC Group	IcSCI Group	NI Group
Primary objective*	CHWs have usable and quality essential medicines available when needed for appropriate treatment of pneumonia and other common diseases of childhood	63% of CHWs had all 5 products in stock on DOV†, significantly better than comparison group (*P* < 0.001)	45% of CHWs had all 5 products in stock on DOV†	38% of CHWs had all 5 products in stock on DOV†
Product flow*	Clear procedures and processes for inventory management, distribution, and storage exist and are executed as expected	100% of CCs reported that they picked up products for all CHWs in their cell after every monthly meeting	91% of CCs reported that they picked up products for all CHWs in their cell after every monthly meeting	N/A
		93% of CHWs reported that they received products regularly	93% of CHWs reported that they received products regularly	85% of CHWs reported that they received products regularly
		95% of CHWs reported that they received products from CCs	93% of CHWs reported that they received products from CCs	26% of CHWs reported that they received products from CCs (majority receive from CHW Supervisor – 63%)
Data flow*	Logistics (consumption and stock levels) data are available and usable for supply chain decision making, management, monitoring, and problem solving	81% CHWs reporting on time	86% CHWs reporting on time	N/A
		97% of HCPM have copies of any resupply worksheets submitted by CCs at the last monthly meeting	92% of HCPM have copies of any resupply worksheets submitted by CCs at the last monthly meeting	N/A
			% of CCs who presented complete RSWs without any calculation errors during monthly health center meetings improved from average 77% for the three districts in the first quarter, to 98% in the final quarter (source: IcSCI indicators database)	N/A
		CCs had key QC tools completed with data collected to use for quality improvement: • 93% CCs could show the bar graph for last month of QIT • 91% of CCs could show the tally sheet for last month of QIT • 97% of CCs who could show tally sheets and bar graphs had agreement between the two records for the last month of the intervention		N/A
		83–98% of CHWs had stock cards on day of visit for amoxicillin, ORS, zinc, Primo Rouge, and RDTs, significantly better than comparison group for same products	83–95% of CHWs had stock cards on day of visit for all five iCCM products, significantly better than comparison group for same products	65–83% of CHWs with stock cards on day of visit for all five iCCM products
		36% of CHWs had accurate stock card for all 6 product	33% of CHWs had accurate stock card for all 6 products	18% of CHWs had accurate stock card for all 6 products
**Effective people**	Management processes and skills exist; Teamwork takes places across multiple levels, using data for problem solving; CHWs are motivated and recognized for SC accomplishments	High levels of competency were found in completing RSWs; 80% of CCs were able to enter correct quantities required	High levels of competency were found in completing RSWs; 86% of CCs were able to enter correct quantities required	N/A
		77% of HCs could show their completed Q3 action plan	All districts showed significant improvements in 3 key SC indicators across 4 implementation quarters (source: IcSCI indicators database)	N/A

ACT – artemisin–based combination therapy, CC – Cell Coordinator, iCCM – community case management, CHW – community health worker, DOV – day of visit, HCPM – Health Center Pharmacy Manager, IcSCI – Incentives for Community Supply Chain Improvement, QC – Quality Collaboratives, NI – non intervention comparison group, ORS – oral rehydration solution, Q3 – 3rd quartile, QIT – Quality Improvement Team, RDT – rapid diagnostic tests (malaria), RSW – resupply worksheet, SC – supply chain

*Comparison group data available for Primary Objective, and for select Product Flow and Data Flow data points, other data points in the table relate specifically to the interventions and are not relevant in the comparison group.

†Products include amoxicillin, 150 mg, ORS, zinc 10mg, Primo Rouge (ACT 1 × 6), Primo Jaune (ACT 2 × 6).

**Product flow.** The RSP intervention was meant to shift responsibility for product collection away from CHWs to the CCs, while streamlining resupply by reducing inefficiencies in travel and congestion at health center pharmacies. Results presented in **Table 6** suggest improvements in product flow, with 100% of CCs in QC and 91% in IcSCI reporting that they picked up products for all CHWs in their cell after every monthly meeting and 95% of CHWs in QC and 93% in IcSCI reporting that they received products from CCs. Looking across groups at follow up, significantly more CHWs from both intervention groups (93% for QC and 93% for IcSCI) reported regularly receiving medicines and health products to treat sick children, compared with the non–intervention group (85%; *P* < 0.05). Data from the IcSCI indicators database showed that the percentage of CCs who collected needed products for their cell after the HC meeting was 96% during the last quarter of the testing period.

Qualitative findings also supported an improvement in product flow. The RSPs bring order to the resupply process, as described by a CHW Supervisor from the IcSCI group who offered, *“Before [RSPs], there was no proper procedure and CHWs could come to the pharmacy any time to request for products. It was total chaos.”* Another supervisor from the QC group explained, *“[Prior to RSP implementation] it was jungle law and often many CHWs went away empty–handed. The quick ones took away too many drugs, which kept expiring in the community…As a result of all this confusion, [we] were in constant conflict with pharmacy staff…now…total harmony reigns between us and the pharmacy staff. No unnecessary drugs are expiring.”* Related to enabling evidence–based decision making, a supervisor from the QC group stated, *“Using the fiche de calcul [magic calculator] helps the health centers to know exactly how much products are required. Without it everyone would be lost because the CHWs can demand anything, leading to wastage and misuse of scarce resources. It helps the CC to know who needs what and when.”*

**Data flow.** Follow up results showed that RSPs led to better data capture through stock cards and data flow from the CHW to the CC to the health center pharmacy. In terms of stock card accuracy, 36% of CHWs in the QC group kept accurate stock cards for all six products, significantly better than 18% in the NI group (*P* < 0.05), while 33% of CHWs in the IcSCI group followed close behind with keeping accurate stock cards. Moreover, “reporting rates” were high, with 97% of Health Center Pharmacy Managers in the QC group and 92% in the IcSCI group keeping copies of RSWs from all or some cells associated with their health center from the most recent month (prior to the survey). Cell Coordinators reported high rates of meetings (where data are captured and calculated); in both groups, 100% of CCs reported that they held meetings each month and 92% reported that all CHWs bring all of their stock cards to meetings. Only 11 of 136 (7%) CCs trained reported problems using RSWs.

**Effective people.** SC4CCM supported implementation of the QC intervention and QIT meetings took place regularly with good attendance, and use and availability of tools were high. However, this by itself does not illustrate the intervention’s effect on product availability. The most useful intermediary data bridging the gap between occurrence of meetings and improved product availability came from the FGDs. The FGD findings suggest that QCs enhanced planning and teamwork, as one Pharmacy Manager offered… “*The QIT has built such a good relationship along the entire chain. For me the biggest prize has been to learn how to work on a plan and be able to achieve it every month.*” FGD participants also underlined the motivating effect of the QC, one supervisor explained, *“Learning sessions were very important. Each group would exhibit their achievements and challenges. This allowed us to learn from those who had faced a similar challenge in the past and how they solved it.”*

In the IcSCI group, three of nine incentives indicators showed strong evidence of significant SC improvements between Q1 and Q2, and continued high performance across the group over the remaining three quarters of the test period, as would be expected for an effective incentive scheme. Results from the IcSCI database show the proportion of CHWs with stock cards for iCCM products where *physical inventory matches stock card balance* for all on the DOV, increased from 86% in Q1 to 96% in Q4 (n ranges 3157–3201 CHWs visited in the three intervention districts each quarter, over four quarters). We found an improvement of 7.03% between Q1 and Q2 for this indicator across the three districts, *P* < 0.001, 95% CI [5.5–8.57%]. The proportion of CCs who presented complete RSWs *without any calculation errors* during monthly health center meetings, in the past quarter, rose from 77% in Q1 to 98% in Q4 (n same as above). Results from the three districts show a 13.43% difference between Q1–2, *P* < 0.001, 95% CI [8.8–18.2%]. The proportion of CHWs who have *at least one treatment for a five–year–old child in stock*, for each iCCM product on the DOV, rose from 79% in Q1 to 92% in Q4 (n same as above). Results from the three districts show a 14.06% difference for this indicator between Q1–2, *P* < 0.001, 95% CI [12.9–15.2%].

**Comparing Intervention Packages.** In comparing the results of QCs and IcSCIs, it is possible to consider the additional value of the teamwork component of the effective people element. While QCs performed slightly better than IcSCIs in key supply chain performance indicators (eg, reporting completeness, stock card accuracy, and six–month stock out rates) there were no significant differences, and both performed better than the NI group. However, there were differences in the overall impact on product availability.

The follow up survey found significantly greater availability among CHWs of all five iCCM products in stock on the DOV in the QC group (63%) compared to NI group (38%; *P* < 0.001), and non–significantly greater availability in the IcSCI group (45%) compared to NI group (38%). A significant decline was detected since baseline for this measure in the NI group (from 58% to 38%; *P* < 0.01).

Further analysis of in–stock data using DiD analysis showed a highly significant improvement (*P* < 0.001) in the QCs group compared with the NI group for the key composite indicator of all five iCCM products in stock on DOV. The DiD detected significant improvements in availability for all products individually (*P* values range from <0.05 to <0.01 for ORS, zinc, and ACT 1 × 6), with the exception of amoxicillin and ACT 2 × 6. In the IcSCI group, a significant result was detected only for one product, ACT 1 × 6 (*P* < 0.05), but no results for other individual products or the composite indicator.

### Ethiopia

The supply chain Ready Lessons and Problem Solving approach was *not* implemented exactly as designed; however it still proved to be a rapid, affordable and effective way to build a foundation in supply chain knowledge and skills for CHWs. As previously mentioned, the project’s activities only targeted the effective people element, and only laid the foundation for two components of this element – management and teamwork. As this was primarily a training intervention, the aim was not to measure product availability but to determine whether the training led to competency, setting a foundation for improved practices supporting product flow and data flow. Therefore the follow up survey focused on whether health center staff could train CHWs opportunistically and affordably and in a way that built skills. Survey results are largely limited to coverage and competency of the CHWs six months after the Ready Lessons were introduced. However other supply chain indicators are provided in **Table 8** but the results are mixed and not always a reflection of the intervention. Further interventions are being tested to determine what is required to fully achieve all three elements and improve product availability significantly (results are expected in late 2014). **Table 8** summarizes follow up survey results for Ethiopia.

**Table 8:** Summary of quantitative follow up survey results, Ethiopia

	Definition	Intensive Group (Ready Lessons, Problem Solving, Follow Up)	Non–intensive Group (Ready Lessons, Problem Solving, No Follow Up)	Comparison (OJT) Group
**Primary objective**	CHWs have usable and quality essential medicines available when needed for appropriate treatment of pneumonia and other common diseases of childhood	27% of CHWs had all 5 products* in stock on day of visit	36% of CHWs had all 5 products* in stock on day of visit	36% of CHWs had all 5 products* in stock on day of visit
**Product flow**	Clear procedures and processes for inventory management, distribution, and storage exist and are executed as expected	61% of CHWs report they are supposed to receive products monthly	39% of CHWs report they are supposed to receive products monthly	23% of CHWs report they are supposed to receive products monthly
		99% of CHWs report getting their health products from the health center, 11% from district health office, 4% from NGO (multiple responses allowed)	94% of CHWs report getting their health products from the health center, 5% from district health office, 10% from NGO (multiple responses allowed)	93% of CHWs report getting their health products from the health center, 22% from district health office, 25% from NGO (multiple responses allowed)
**Data flow**	Logistics (consumption and stock levels) data are available and usable for supply chain decision making, management, monitoring, and problem solving	87% of CHWs trained know they are supposed to submit the HPMRR† every month to the higher level	59% of CHWs trained know they are supposed to submit the HPMRR† every month to the higher level	14% of CHWs trained know they are supposed to submit the HPMRR† every month to the higher level
**Effective people**	Management processes and skills exist	84% of CHWs were trained in IPLS	62% of CHWs were trained in IPLS	17% of CHWs were trained in IPLS
		65% of CHWs completed the most important data for the bin card correctly	59% of CHWs completed the most important data for the bin card correctly	62% of CHWs completed the most important data for the bin card correctly
		36% of CHWs completed the most important data for the HPMRR† correctly	29% of CHWs completed the most important data for the HPMRR† correctly	25% of CHWs completed the most important data for the HPMRR† correctly
		68% of HEWs (I and NI) report participating in a problem solving (PS) session during monthly meetings	26% of HEWs (I and NI) report participating in a problem solving (PS) session during monthly meetings	N/A‡
		85% HC staff report conducting IPLS PS sessions with HEWs	53% HC staff report conducting IPLS PS sessions with HEWs	N/A‡

ACT – artemisin-based combination therapy, CHW – community health worker, IPLS – Integrated Pharmaceutical Logistics System, HC – Health Center, HEW – health extension worker, I – intervention, N/A – not applicable, NI – non intervention, NGO – Non Governmental Organization, OJT – on the job training, ORS – oral rehydration solution, RUTF – ready-to-use therapeutic food

*Products include cotrimoxazole 120 mg, either ACTs (1 × 6 and 2 × 6), ORS, zinc and RUTF.

†HPMRR refers to the Health Post Monthly Report and Request form.

‡Problem solving was not part of the intervention package for the comparison group.

**Product flow and data flow**. Product flow and data flow indicators did improve across all groups; however, the intensive group, where the effective people component was implemented to a greater extent through follow up support, showed better results in terms of CHWs’ knowledge: more CHWs knew they should receive products monthly and that they should submit a HPMRR form each month to the higher level. Data are not available on if this knowledge was translated into practice. The process of receiving products from the health center appeared more standardized across groups with more CHWs receiving products from health center (as per IPLS) however this was not necessarily due to the intervention as there was a policy change at national level directing CHWs to collect salaries and products from the nearest health center.

**Effective people.** The follow up survey results show that six months after training health center staff, the number of CHWs trained in supply chain had increased five–fold and CHW competency and knowledge had improved. In the intensive group (I), where health center staff received support from the project and higher levels to organize trainings and conduct follow up [15], 84% of CHWs surveyed had been trained in supply chain, compared with 62% in non–intensive (NI), and 17% in the comparison group (C). All increased from the baseline (11%). CHW knowledge improved, with 87% of CHWs in the intensive group, 59% in non–intensive group, and 14% in comparison group knowing to submit reports to health centers, compared to 5% at baseline. CHW competency varied by task, being higher for a simpler task of completing a bin card correctly (65% intensive, 59% non–intensive, 62% comparison), and lower for the most complicated skill of completing the HPMRR form (36%, 29%, and 25% respectively) [16]. The latter modest performance scores were, in fact, a dramatic improvement over baseline (0%) and would likely improve further over time with practice and targeted supportive supervision. Ready Lessons had improved the skills of CHWs laying the groundwork for improving management processes.

Sixty eight percent of CHWs in the intensive group reported participating in a Problem Solving session during monthly meetings compared to 26% in the non–intensive group, the team building component of this intervention (problem solving was not part of the intervention package for the comparison group). At the health center level, 85% of HC respondents in the intensive group and 53% in non–intensive group reported conducting Problem Solving sessions with CHWs. However, despite slow rollout of the Problem Solving sessions, when CHWs were asked about their usefulness, CHWs from all regions stressed that the problem solving was very important for strengthening the IPLS at the CHW level.

**Comparing Intervention Packages.** As seen in **Table 8**, improving CHW knowledge of the reporting and resupply processes was not sufficient to have an impact on product availability at community level. The results show that in the comparison group where the least number of CHWs were trained, the product availability was higher. More CHWs in this group reported receiving products from an NGO and it is therefore likely due to the presence of kits which were being distributed ad hoc and not in response to need. When considering the other results and comparing the different approaches to making people effective we see that the intensive group which received more follow up and had a larger number of Problem Solving sessions did better for many of the indicators, suggesting that achieving the effective people element requires more than just training staff.

## Discussion

The findings from all interventions in the three countries suggest that the greatest supply chain benefits are realized when all three elements (product flow, data flow, and effective people) are in place and working together. This is most clearly demonstrated by the benefits of synergy on the supply chain as demonstrated by the EM and QC results; these interventions brought together product flow, data flow, and effective people to achieve the greatest improvements in supply chain to the community level. The Rwanda IcSCI intervention, that also brings all three elements together but does not directly address the multi–level team work component of effective people, showed less detectable improvements in SC performance and product availability compared to the QC. This suggests that the three elements work best together when all components related to each element are part of the intervention design. Results from the EPT group in Malawi where data flow and effective people were only partially addressed and Ethiopia where effective people was only partially addressed, further suggest that when only one or two elements are present, only minor or incremental benefits are observed, and effectiveness of the supply chain – as measured by improvements in supply chain performance, supply reliability or product availability – are not affected. Quantification and national product availability proved to play an important role in determining product availability at the community level – pointing to the importance of implementing community health supply chain improvements within the context of the overall supply chain.

### Product flow, data flow and effective people: interconnected elements

Product flow and data flow, though two distinct elements of the CHSC Framework, need to be deliberately linked, aligned and synchronized during intervention design to ensure that the right data are collected and made available to the right person (eg, the person resupplying a CHW needs access to consumption and stock on hand data to determine resupply quantities) who then uses it to make informed decisions on resupply/product flow. The inter–relationship between these two elements are further strengthened and sustained when levels of the system supplying the data see the associated benefits, such as products flowing to them based on demand. In addition, data and product flow must also align with management practices and workflows, as part of the effective people element, to realize maximum benefits in the SC, as seen in the case of Rwanda QCs and Malawi EM where streamlined data flow combined with a structured mechanism for reviewing and using data made people more effective and involved in managing product flow thus resulting in improvements in supply reliability and supply chain performance.

The “gold standard” is an EM–like solution that combines a demand–based system with a real time reporting system, such as a mHealth system, that allows inventory data to be available at all levels of the system simultaneously to enable rapid decision making and response as well as activities such as performance monitoring, management and quantification. Although the RSPs in Rwanda included a demand–based system that resulted in effective data flow and product flow between the community level and resupply point, the manual nature of the data flow system prevented CHW logistics data from being immediately available at levels beyond the CHW resupply point to enable effective community supply chain performance monitoring and management by district and central level managers.

### Effective people

The effective people element of supply chain interventions – despite its potential for reinforcing product and data flow and improving community health supply chain practices – is the element most often left out, in part because of the required time investment and challenges to monitoring and measurement. Additionally, because CHWs generally are at the last mile they can be isolated from the main health system. Program design to support effective people can reduce perceptions of isolation by making supply chain performance and supply reliability a joint goal amongst facility–based staff and CHWs. However, this requires ongoing commitment at multiple levels to ensure that CHWs, at the end of the supply chain, receive routine support and feedback from managers who are close to them in the chain. The qualitative data for the EM and QC interventions best demonstrate how and why the effective people component is so important in enhancing results; DPATs and QITs strengthened linkages across multiple levels of the health system, enhanced communication and understanding of tasks, and established common goals and a collective responsibility for achieving results, while motivating CHWs who performed well.

In Malawi, the difference between the EM and EPT groups on key supply chain performance indicators such as complete reporting, reduced lead times, and stock out rates, can be attributed to the DPAT component in the EM intervention, underlining the importance of the effective people component. In Rwanda, the RSP intervention rationalized data and product flow and the QC and IcSCI groups addressed management and motivation each in slightly different ways. The IcSCI intervention included the management and motivation components of effective people which accelerated the immediate uptake and utilization of the data and product flow process (RSPs) and contributed to behavior change of CHWs towards improved performance of supply chain tasks. However the difference in findings between QC and IcSCI demonstrates the added benefit of the formalized team component in the QC group, where greater improvements in supply chain performance and product availability could suggest that this is related to the effects of multi–level teams working together to identify and solve problems related to community supply management, rather than the single–level nature of cooperatives as teams. The significant improvements detected by the DiD for the QCs intervention also establish evidence of this as a successful method for improving iCCM product availability at the CHW level.

In Ethiopia, an important finding was that using health center staff to train CHWs in basic supply chain knowledge and skills by incorporating lessons into existing activities can significantly improve training coverage in a short period of time and is affordable since it doesn’t require extra travel or allowances. However, although the Ready Lessons/Problem Solving intervention yielded a reasonable improvement in supply chain competency levels, it wasn’t sufficient to affect supply chain performance or improve product availability. In Ethiopia, the intervention mainly addressed management, while structured problem solving increased contact between CHWs and HCs, but did not establish a sense of a team with common goals or involve district levels to help with more complex problems, as was seen in Malawi and Rwanda, two differences that might explain limited achievements. Essentially, the Ethiopia experience demonstrates that *within* the effective people component, all three components must work together – management, teamwork, and motivation – so that optimal results are achieved.

### Quantification and national coordination

As the foundation to the product flow – data flow – effective people cycle, quantification and national coordination are important central–level activities that support community–level product availability. If community–level needs are not carefully considered and estimated in national quantifications and procurements, CHWs will likely suffer the most from shortages and expiries since they are at the end of the supply chain, regardless of how well the lower level supply chain functions. Therefore, to see optimal product availability at the community level and realize the benefits of designing and implementing interventions using the CHSC framework, sufficient quantities of products need to be available at higher levels. This requires the flow of data from the community level as well as coordination with data from higher levels where the same products are used and careful oversight of stock levels, considering total system demand for each product. Some of these challenges are overcome by using unique products as was seen in Rwanda, as it is easier to quantify for iCCM as a stand–alone program and ensure that products are not used up before they arrive at the community level.

Coordination between MOH programs, donors, and procurement units is also important for ensuring that available resources are used efficiently and that products used by multiple levels/programs are sufficient for all intended uses. The level of coordination required is often difficult to achieve, especially in countries where procurement for community–level products spans multiple programs and donors. In Rwanda, for instance, follow up results for product availability were aided by strong coordination at the MOH level and the use of unique products at the community level which made it easier to estimate CHWs’ needs and ensure that products were available at resupply points for community use. On the other hand, Malawi experienced an economic crisis and currency devaluation during the test period, which impacted the distribution and availability of supplies. Given the sudden lack of funds available for functions that typically fall under the government’s purview, partners stepped in to support parallel supply chains and procure and distribute products outside the system that the interventions were meant to strengthen. Simultaneously, government budgets for procurement of essential medicines were dramatically reduced and uncertain, making central–level coordination and planning very difficult. Intervention results in Malawi made clear that the political and economic environment as well as the national product availability environment and distribution mechanisms play important roles in determining product availability at the community level – pointing to the importance of implementing community health supply chain improvements within the context of the overall supply chain, where quantification and national coordination take place regularly and effectively, and the overall supply chain is characterized by strong organization/leadership. Aligning management, motivation, and teamwork with coordination and routine quantification creates a system with improved availability of data and products when and where needed.

## CONCLUSIONS

In addition to an enabling political and economic environment, there are many factors necessary for ensuring continuous product availability at the community level. Assuming the presence of sufficient capacity and funding to procure products on a regular basis, an in–country distribution system works best when three key elements (product flow, data flow, and effective people) are included in system design. The way these are implemented may look different in each setting, as they were designed with the local context and longer term scale and sustainability in mind [17]. However in all countries the common finding was that intervention designs need to ensure that data flow and product flow processes are streamlined, aligned, and reinforced by an effective and supportive workforce that is organized into multi–level teams with common objectives and structures for supervision, that use data to improve supply chain performance and communicate regularly to promote product availability at the community level.
